# Does the Risk Premium Differ Between Women Engaging in Commercial and Transactional Sex? Evidence From Urban Cameroon

**DOI:** 10.1002/hec.4964

**Published:** 2025-06-13

**Authors:** Rebecca G. Njuguna, Henry Cust, The POWER Team, Aurélia Lépine

**Affiliations:** ^1^ Health Economics Research Centre Nuffield Department of Population Health University of Oxford Oxford UK; ^2^ Centre for Global Health Economics Institute for Global Health University College London London UK; ^3^ Sanford School of Public Policy Duke University Durham North Carolina USA

**Keywords:** commercial sex, HIV, risk premium, transactional sex, unprotected sex

## Abstract

Female sex workers (FSWs) can receive a premium for engaging in unprotected and other risky sexual behaviours. Women engaging in transactional sex, defined as ‘non‐commercial sexual relationships motivated by the implicit assumption that sex is exchanged for material support’, are thought to share similar economic incentives as women engaging in commercial sex. Using a panel of up to six sex acts from longitudinal datasets stratified by FSWs and women engaging in transactional sex in Cameroon, we provide evidence consistent with literature of a 30% condomless risk premium for FSWs. We then provide the first empirical evidence of a discount for condomless sex of 14% for women engaging in transactional sex. Qualitative analysis offers two explanations for this surprising finding, first a lack of HIV awareness among women engaging in transactional sex, and second, that risky sex acts are a demonstration of investment of trust in relationships and represent unobservable exchange of value. Given the larger number of women engaging in transactional relationships compared to FSWs in sub‐Saharan Africa, and their lower awareness of HIV risks, this finding offers a significant explanation for the disproportionate burden of HIV incidence among adolescents and young women in sub‐Saharan Africa.

## 1 Introduction

Women involved in sex work face a heightened risk of HIV infection, both in sub‐Saharan Africa and globally, partly driven by the economic incentives tied to the “risk premium” for unprotected sex and other high‐risk behaviours. Studies indicate that this risk premium serves as a mechanism for consumption smoothing in the face of economic shocks (Cust et al. 2021) particularly for women in relative poverty. The ability to earn money quickly through the risk premium makes it an attractive option in times of financial need. However, this premium can undermine the effectiveness of pre‐exposure prophylaxis (PrEP) through risk compensation behaviours (Cassell et al. 2006; Eaton and Kalichman 2007; Quaife et al. 2021). Additionally, women engaging in transactional sex—defined as “non‐commercial, non‐marital sexual relationships motivated by the implicit expectation of material support or other benefits”—experience up to a 50% higher HIV risk compared to the general population (Stoebenau et al. 2016; Wamoyi et al. 2016; Dunkle et al. 2004; Kilburn et al. 2018; Ranganathan et al. 2016) and point to a number of risk factors, including age‐disparate relationships (Ranganathan et al. 2020; Luke 2003; Potgieter et al. 2012; Cockburn et al. 2010; Luke 2005), violence (Choudhry et al. 2014; Cluver et al. 2011; Jewkes et al. 2006; Okigbo et al. 2014), reduced bargaining power (Ranganathan et al. 2017), multiple concurrent partnerships (Moore et al. 2007; Steffenson et al. 2011; Phillips‐Howard et al. 2015; Okigbo et al. 2014) and inconsistent condom use (Luke 2005; Luke et al. 2011) among others (Stoebenau et al. 2016).

Although women in transactional sex may face similar economic incentives to engage in risky sexual behaviours as female sex workers (FSWs), no research has yet explored whether the risk premium affects both populations in a similar way. This paper has two primary objectives: First, to determine whether a risk premium exists for women engaging in transactional sex, and if so, to quantify it. Second, we aim to compare this premium to that of FSWs operating in the same context, Yaoundé, the capital of Cameroon. To achieve this, we use data from a panel of up to six sex acts collected across three waves of observations from a cohort study. The waves were equally stratified between women involved in commercial sex and those in transactional sex. By leveraging the panel nature of the data and controlling for participants fixed effects. We estimate risk premiums separately for both groups, thereby minimizing the influence of time‐invariant confounders.

Previous literature has consistently documented the presence of a risk premium for unprotected sex among FSWs globally (Quaife et al. 2019; Rao et al. 2003, 200; Torre et al. 2010; Adriaenssens and Jef 2012; Muravyev and Talavera 2018; Egger and Lindenblatt 2015; Cunningham and Todd 2014). In low‐ and middle‐income countries (LMICs), the highest estimates of the unprotected sex premium were reported as 350% in the Democratic Republic of Congo (Ntumbanzondo et al. 2006), 136% in Kenya (Jakubowski et al. 2016), and 81% in Bangladesh (Islam and Russell 2012). More modest estimates include 23% in Mexico (Gertler et al. 2005), 13% in Ecuador (Arunachalam and Shah 2013), and 24% in Busia, Kenya (Manda 2013). The lowest estimate, at 9.3%, was found by Robinson and Yeh (2011) in Kenya, with the authors attributing this lower figure to the inclusion of informal FSWs—likely women involved in transactional sex—in their sample.

The estimation of the risk premium among FSWs may have been hindered by the inclusion of women engaging in transactional sex, as many studies tend to conflate the two activities. While both involve sexual relationships with material benefits, transactional sex and commercial sex are distinct in their motivations, structures, and forms of compensation. First, while commercial sex is primarily economic with formalized, closed exchanges, transactional sex is more relationship‐oriented, informal, and open‐ended (Duby et al. 2021; Wamoyi et al. 2016). In transactional sex, the terms of exchange are rarely explicit, and compensation is not necessarily immediate or tied to specific sexual acts. Second, unlike commercial sex where compensation is typically monetary, in transactional sex, compensation may be delayed and can include non‐monetary forms, such as social status, services, or gifts, as well as non‐material benefits like companionship or emotional support (Wamoyi et al. 2019).

This variability complicates the measurement of value associated with each sexual act. Third, women involved in commercial sex usually identify as sex workers and refer to their partners as “clients,” while those engaging in transactional sex do not identify as sex workers, instead referring to their partners as “boyfriends” or “sugar daddies.” The motivations within these relationships often include emotional intimacy alongside material or financial support, which suggests that the male partners may have different motivations compared to typical clients of FSWs. There is a notable lack of literature on male partners in commercial sexual relationships, and even more so for the partners of women involved in transactional sex (often referred to as sugar daddies). Theoretically, the characteristics and preferences of these male partners are likely distinct from those of typical clients of FSWs. Some literature does indicate a significantly higher risk of infection among FSWs who identify as “non‐professional” or “private” FSWs, which may overlap with women involved in transactional sex, compared to “regular” FSWs (Nagot et al. 2002; Lépine et al. 2025). Qualitative interviews conducted with women engaging in transactional sex during our data collection also suggest that payoffs were responsive to condom use. This responsiveness hints at the importance of the risk premium in explaining the elevated HIV risk among women in transactional sex.

Our findings reveal that women engaged in commercial sex earn a premium of up to 30% for unprotected sex, consistent with existing literature. However, unexpectedly, for women involved in transactional sex, the risk premium is negative, with a discount of approximately 14% for unprotected sex. We hypothesize that this discount may stem from the non‐explicit nature of payments, as well as the dynamics of preferences and trust within transactional sex relationships. Our results further suggest that engaging in unprotected sex within these relationships increases the likelihood of receiving no explicit compensation for the act. While we provide tentative explanations for this surprising outcome, further research is necessary to better understand the underlying causes.

This discount for women involved in transactional sex, and the associated factors explored in this paper, could provide important insights into the disproportionately high burden of HIV experienced by adolescent and young women in sub‐Saharan Africa. Historically, gender disparities in HIV have been attributed to greater biological susceptibility, exacerbated by co‐infections with sexually transmitted infections (STIs) (Fleming and Wasserheit 1999; Oster 2005). However, recent evidence highlights the significant role of socio‐economic factors—particularly poverty and gender inequality—in driving these disparities (Lépine et al. 2023; Cust et al. 2021; Magadi 2011), to which this paper contributes.

The paper begins with a detailed description of the dataset and descriptive statistics for women engaging in both commercial and transactional sex. Next, we outline the empirical strategy employed to estimate the condomless sex premium for these two subgroups. We then present the results of our primary analysis, followed by robustness checks. Finally, we discuss the broader implications and limitations of our findings before concluding the study.

## 2 Setting and Data

The data for this paper is drawn from the randomized controlled trial (RCT) titled Protecting Women from Economic Shocks to Fight HIV in Africa (POWER) (Lépine, Szawlowski, et al. 2024). The study recruited 1508 adolescents and young women involved in both commercial and transactional sex between June 2021 and March 2022 in Yaoundé, Cameroon. Our analysis includes all three waves of data from the RCT, incorporating the baseline data and the control group data at both midline and endline. Women randomized to the treatment group were provided with family health insurance as a form of risk‐coping strategy. Given that the intervention aimed to mitigate incentives for risky behaviours, women in the treatment group were excluded from the analysis.

HIV prevalence in Cameroon is estimated at 3%, making it one of the highest in West and Central Africa (UNAIDS 2021). Moreover, the country experiences significant gender disparities in HIV, with women bearing twice the prevalence rate of men (CAMPHIA 2018). This disparity is even more pronounced among younger women aged 15 to 24, where HIV prevalence is triple that of their male peers (CAMPHIA 2018).

Commercial and transactional sex have been identified as key drivers of the country's large gender disparity in HIV. The estimated HIV prevalence among FSWs in Cameroon stands at 24.3%, substantially higher than the national population average of 3% (UNAIDS 2021). While selling sex is illegal in Cameroon, it is tolerated, particularly in urban and tourist areas, with Douala and Yaoundé being the major hubs (Billong et al. 2019). It is estimated that approximately 2% of adult women in the country engage in commercial sex as their primary source of income (Billong et al. 2019). Additionally, a larger proportion of young women engage in transactional sex, often due to peer pressure, to obtain certain social status, connections to build specific social networks, and acquire luxurious items, which predisposes them to higher risks of HIV infection (Chatterji et al. 2005).

### 2.1 Recruitment and Data Collection

Identification of participants was done in collaboration with community‐based organisations (CBOs) providing services to women engaging in commercial and transactional sex in Yaoundé. Recruitment of participants was done using a respondent‐driven chain‐referral sampling model akin to a snowball methodology. Through the CBOs networks, initial participants (seeds) were identified and recruited and if willing and able, were given invitation cards containing study contact information to distribute and recruit up to three members in their social network (nodes). The nodes were in turn asked to recruit other three members of their network. The study staff explained the study information and eligibility criteria to the selected seeds and nodes before asking them to invite members. The snowballing technique continued until the intended sample size was achieved. Data were collected at three points, baseline, midline (6‐month follow‐up) and endline (12‐month follow‐up). Data were monitored during this period to assess whether women transitioned from one status to another, that is, from commercial to transactional sex work and vice versa. Throughout the period, only one person changed from transactional to commercial and was dropped from the analysis.

### 2.2 Eligibility Criteria for Participants and Ethics Approval

Females aged 15 years or older who were engaging in transactional or commercial sex, who had at least one economic dependent, tested negative for HIV, and were unmarried were eligible to participate in the study. Ethics committees at University College London and the National ethics committee in Cameroon provided ethics approval. Participation was voluntary; respondents gave informed written consent or asset for minors in addition to parental consent and were reimbursed for their transport costs and time.

Data were collected across three waves: biobehavioural surveys were conducted at baseline and were used to collect socio‐economic and risky behaviours for women engaging in commercial and transactional sex. The surveys were conducted via face‐to‐face interviews by trained and experienced local interviewers and took approximately 1.5 h per participant. Information collected from both groups of women included participants' individual characteristics such as age, marital status, education level, number of children and economic dependents, period in sex work or transactional sex, and income earned from the sex transactions. Additionally, information on sexual behaviours and client/sugar daddy characteristics during the respondents' last two sex acts with their last and penultimate clients were collected. This included the amount received per sex act, type of sexual activities performed (unprotected, vaginal, anal, and oral sex that were not mutually exclusive), duration of sex acts, and condom use. There were some differences in these questions (e.g., payment type) between commercial and transactional sex, but the same questions and wording were used where possible.

### 2.3 Survey, Risky Sexual Behaviours and *Colorbox* Method

Given the sensitivity bias arising in collecting data on unprotected sex (Lépine et al. 2020; Lépine and Treibich 2020; Chuang et al. 2021; Treibich and Lépine 2019; Lépine, Treibich, et al. 2020), multiple data collection methods were implemented in this study, direct questioning and the *Colorbox* method (Lépine et al. 2024; Valente et al. 2024; Lepine et al. 2024). The direct method involved directly asking questions such as (“Did you use a condom during sex with your client?”) as part of the information collected. According to the literature, direct questioning during face‐to‐face interviews may be prone to misreporting due to possible social desirability bias (Lépine, Treibich, et al. 2020). Literature shows that indirect elicitation methods reduce the under‐reporting of sensitive behaviours as they eliminate the respondents' fear of being judged or discovered. A study conducted to compare direct and indirect methods used to collect data on condom use among FSWs in Senegal found a 17% overestimation of condom use if questions were asked directly (Treibich and Lépine 2019). In addition to the direct method, the Colorbox method was employed in this study to ensure participant anonymity when providing responses to sensitive questions. The Colorbox method involved using PIN codes linked to colors, which were concealed from the interviewers, to collect participants' responses regarding risky sexual behaviours. This method was specifically applied to elicit information on condom use and anal sex. As such, the direct method estimates were used in the primary analysis, while the Colorbox method estimates served as a validity check for the premium estimates related to condom use and anal sex. Further details on the design and implementation of the Colorbox method can be found in Appendix A.

### 2.4 Descriptive Statistics

Table 1 gives the summary statistics for the two groups of our sample: women engaging in commercial (*n* = 755) and transactional sex (*n* = 753) in Yaoundé, Cameroon. The final column includes the statistical difference between the two groups, telling us that these two sets of women have different characteristics. On average, FSWs were slightly older than women engaging in transactional sex, with median ages of 28 and 23 years, respectively. Notably, the proportion of women engaging in transactional sex who were below 20 years was significantly higher than the number of FSWs.

**TABLE 1:** Descriptive statistics for women engaging in commercial and transactional sex with differences.

Characteristics	Commercial sex (*n* = 755)		Transactional sex (*n* = 753)		Difference
	Obs	Mean (SD)/%	Obs	Mean (SD)/%	*p*‐value
Age (years)	749	30.23 (9.15)	752	24.40 (5.95)	< 0.001***
Under 20 years of age	749	10%	752	26%	< 0.001***
Experience in sex work (months)	755	53.09 (50.17)	752	36.37 [37.10]	< 0.001***
Want to quit sex work	752	94%	744	65%	< 0.001***
Sex as main income source reason for not quitting	81	89%	25	52%	< 0.001***
Head of household	754	83%	750	49%	< 0.001***
Economic dependants	755	3 (2)	753	3 (1)	0.2597
Number of children	755	2 (2)	753	2 (1)	< 0.001***
Number of occasional clients/sugar daddy (week)	680	4 (3)	752	1 (1)	< 0.001***
Number of regular clients/sugar daddy (week)	725	3 (3)	753	1 (1)	< 0.001***
Number of sex acts (week)	751	12 (9)	753	3 (2)	< 0.001***
Expenditure on health care	755	18,195 (31,211.2)	753	24,755 (83,712)	<0.05**
Earnings (last 7 days) FCFA	755	25,597 (24,211)	753	15,876 (20,313)	< 0.001***
Health status and AIDS knowledge					
Was sick in the (last 30 days)	755	29%	753	35%	<0.05**
Had HIV test in the last 12 months	754	93%	752	55%	< 0.001***
Does not think frequent condom use can prevent HIV	750	30%	752	58%	<0.05**
Do not feel threatened by HIV	754	66%	752	35%	< 0.001***
Other occupation apart from CS and TS					
No other occupation	755	69%	753	57%	< 0.001***
Marital/relationship status					
Never been married	755	89%	753	97%	< 0.001***
Separated	755	7%	753	2%	< 0.001***
Divorced	755	2%	753	0%	< 0.05**
Widowed	755	2%	753	1%	< 0.05**
Currently have a partner/boyfriend	755	59%	751	73%	< 0.001***
Education level					
Primary	736	15%	747	7%	< 0.001***
Secondary primary cycle	736	35%	747	25%	< 0.001***
Secondary second cycle	736	29%	747	37%	< 0.001***
Superior (post bac)	736	20%	747	29%	< 0.001***

*Note: p*‐values are presented in parentheses.

***, ** and * denote statistical significance, at the 1%, 5%, and 10% levels, respectively.

On average, FSWs and women engaging in transactional sex reported to have engaged in sex work or transactional sex for 4 and 3 years, respectively. Most women engaging in transactional sex and sex work started these activities for economic reason, in addition, women engaging in transactional sex cited their own choice and family pressure as another reason for involvement, while many FSWs cited encouragement by friends. In both groups, most women reported having these sexual practises as their only source of income (69% among FSWs and 57% among women engaging in transactional sex). FSWs were more likely to be household heads and, therefore, had a greater burden of earning responsibility. Still, a majority of those wishing to quit transactional sex cannot because of the income, but to a lesser extent than FSWs. This highlights an important reason for the women's choice to continue engaging in these sexual practices, confirming previous evidence that most women in commercial sex, but less than half of women in transactional sex in SSA engage for economic reasons (Cust et al. 2021; Wamoyi et al. 2016; Stoebenau et al. 2016).

FSWs had more sex acts per week (12 vs. 3) than women engaging in transactional sex and a greater number of clients/sugar daddies per week (7 vs. 2). This translates to a greater income from sex work, although some of the transactional sex income may not be quantifiable and not captured in this measure accurately. FSWs were generally better educated and aware of their HIV status, therefore feeling more confident about HIV and less threatened by HIV. Education levels were very different between the two study populations with women engaging in transactional sex better educated than FSWs. 50% of FSWs have secondary primary cycle education or less, compared to 66% of women engaging in transactional sex.

Table 2 summarises the sex acts characteristics. Contrary to average earnings, the price per sex act was higher for the transactional sex group, but only 60% of sexual transactions had a payment associated with them compared to 100% of commercial sex transactions. Condom use was higher for commercial sex, supporting the idea that FSWs are better educated about the threat of HIV and receive HIV prevention services such as condoms. Women in transactional sex had a higher proportion of regular sugar daddys, reflecting the more intimate and longer‐lasting nature of their relationships.

**TABLE 2:** Descriptive statistics of the sex acts captured.

Sex act characteristics	Commercial sex		Transactional sex		
	(*n* = 2374 sex acts)		(*n* = 2420 sex acts)		Difference
	Obs	Mean (SD)/%	Obs	Mean (SD)/%	*p*‐value
Average price per sex act (FCFA)	2095	6585 (8842)	1310	11,045 (25,144)	< 0.001***
Received anything for the sex act	2374	100%	2420	60%	< 0.001***
Sex act used condom					
Self‐reported	2093	88%	2010	53%	< 0.001***
Colorbox method	1799	87%	1856	53%	< 0.001***
Sex act characteristics					
Vaginal	2096	98%	2009	98%	0.509
Oral sex	2091	15%	2009	16%	0.306
Anal: Self‐reported	2095	2%	2010	2%	0.778
Anal: Colorbox	1507	6%	1506	4%	<0.05**
Client/sugar daddy characteristics					
Age (years)	2066	37.6 (8.67)	1982	35.4 (9.11)	< 0.001***
Occasional	2096	49%	2000	17%	< 0.001***
Regular	2096	51%	2000	83%	< 0.001***
More handsome than average	2081	12%	1957	18%	< 0.001***
Richer than average	2059	12%	1956	10%	<0.05**
Has a girlfriend/wife	993	68%	1609	56%	< 0.001***
Other activities					
Client/sugar daddy took drugs before activity	2061	41%	1981	30%	< 0.001***
FSWS took drugs before activity	2091	36%	2008	24%	< 0.001***

*Note: p*‐values are presented in parentheses.

***, ** and * denote statistical significance, at the 1%, 5%, and 10% levels, respectively.

## 3 Empirical Strategy

Risky sex premium estimation was done separately for commercial and transactional sex to allow a comparison of both. Data used in the analyses was longitudinal, containing details of up to two sex acts per participant per wave, i.e. a maximum of 6 sex acts per participant across the three data waves.

To estimate risky sex premium, the log of price per sex act paid by clients or sugar daddies (primary outcome) was regressed on various risky sexual behaviours (unprotected, vaginal, anal – obtained via *colorbox*), controlling for the participants' fixed effects and other sex act‐varying factors such as the types of sex acts that took place (unprotected, vaginal, anal, and oral, not mutually exclusive), if the participant was suffering from STI symptoms at the time of the sex act, some client's characteristics (client age, perceived client wealth, if the client is a regular or occasional one) and the survey wave (baseline, midline, endline and if the sex act was the penultimate transaction or not). For women engaging in transactional sex, if the compensation was not in cash, we asked them to provide an estimate of the monetary value of the goods or benefits they received.

Participant fixed effects were included to control for time‐invariant unobserved heterogeneity that could influence both the likelihood of engaging in risky sex and the price received. For example, if a participant consistently engages in higher‐risk behaviours and commands higher prices due to negotiation skills or physical appearance, these stable but unobserved characteristics are captured by the fixed effects. This approach accounts for individual‐level unobserved heterogeneity, allowing the model to better isolate the causal impact of unprotected sex on the price received.

The estimating equation was expressed as follows.

(1) $$\ln\left(P_{\text{ij}}\right)=\theta +\beta_{1}X_{\text{ij}}+\beta_{z}C_{\text{ij}}+\alpha_{i}+\gamma_{j}+\varepsilon_{\text{ij}}\;;$$

Where $\ln\left(P_{\text{ij}}\right)$ represents the log of the price paid per sex act to participant i for sex act j, θ is the intercept, $X_{\text{ij}}$ is a dummy variable indicating the sex act was unprotected, $C_{\text{ij}}$ are a series of sex‐act level and client‐level characteristics $\alpha_{i}$ is the participants' fixed effects, $\gamma_{j}$ is the wave number fixed‐effect, $\varepsilon_{\text{ij}}$ is the mean‐zero random error. Results tables display changes in logged price given unit changes in explanatory variables. We exponentiate the coefficient to find the exact percentage change for the unlogged price when interpreting our models in the text.

## 4 Results of Risky Premium

Table 3 shows us the premium attached to unprotected sex for women engaging in commercial and transactional sex. Each model is estimated using the first difference fixed effects at the sex act level. Columns reading left to right add sex‐act differing characteristics.

**TABLE 3:** Premium for unprotected sex.

	Commercial sex ‐ log of price			Transactional sex ‐ log of price			
	(1)	(2)	(3)	(4)	(5)	(6)	(7)
If no condom was used	0.264***	0.224**	0.189*	−0.154***	−0.156***	−0.137**	−0.137**
	(0.102)	(0.103)	(0.105)	(0.058)	(0.059)	(0.059)	(0.059)
Sex acts at midline	0.103	0.105	0.220**	0.266***	0.257***	0.145*	0.134*
	(0.100)	(0.099)	(0.108)	(0.069)	(0.071)	(0.074)	(0.074)
Sex acts at endline	−0.024	−0.023	0.046	0.194***	0.196***	0.171**	0.161**
	(0.091)	(0.091)	(0.097)	(0.072)	(0.073)	(0.079)	(0.079)
Penultimate transaction	−0.198***	−0.187***	−0.134**	−0.028	−0.027	−0.023	−0.025
	(0.052)	(0.052)	(0.053)	(0.040)	(0.041)	(0.041)	(0.041)
Sex act characteristics							
Oral sex		0.457***	0.403***		0.040	0.059	0.057
		(0.112)	(0.113)		(0.078)	(0.077)	(0.077)
Anal sex (direct question)		−0.309*	−0.393**		0.023	−0.034	−0.036
		(0.179)	(0.185)		(0.146)	(0.144)	(0.144)
Vaginal sex		0.730*	0.791**		0.163	0.512	0.518
		(0.382)	(0.391)		(0.737)	(0.710)	(0.709)
Client characteristics							
Client age			0.017***			0.031***	0.031***
			(0.005)			(0.005)	(0.005)
Client was rich			0.268***			0.040	0.037
			(0.063)			(0.046)	(0.046)
Client was a regular			0.086			0.085	0.082
			(0.079)			(0.069)	(0.069)
Woman suffering STI symptoms			−0.141			−0.019	−0.019
Whilst With client			(0.157)			(0.079)	(0.079)
Type of payment							
Received cash							−0.190*
							(0.109)
Observations	2060	2054	2001	1279	1274	1229	1229
R‐squared	0.017	0.033	0.063	0.037	0.036	0.116	0.120
Number of women	752	752	744	628	628	617	617
Sex act characteristics	—	X	X	—	X	X	X
Client characteristics	—	—	X	—	—	X	X
Payment type	n/a	n/a	n/a	—	—	—	X

*Note:* Standard errors are presented in parentheses.

***, ** and * denote statistical significance, at the 1%, 5%, and 10% levels, respectively.

After transforming the coefficients, the premium associated with condomless sex for women in commercial sex lies between 21% and 30%, columns 3 and 1, respectively. These findings are consistent with the literature discussed previously. Key client characteristics such as age and perceived wealth are important predictors of price, each additional year of a client leads to a 1.7% increase in the price charged and being perceived as a rich client increases the price paid by 31%.

Most interestingly, however, are the results for women in transactional sex, columns 4–7, where we add the same sex act characteristics, except in the final columns where we add a variable indicating whether cash was received. These models tell us that not only is unprotected sex not associated with a premium, but that sugar daddies receive a discount in the amount they pay for unprotected sex. This discount ranges from 14% to 16%, see columns 4 and 6, respectively. The age of sugar daddies, likely a proxy for, and highly correlated with wealth or income, is also crucial in determining the price, with a 1‐year increase in their age equating to around a 3.1% increase in the price paid for a sex act.

### 4.1 Alternative Forms of Payment for Women in Transactional Sex

One reason for which women in transactional sex provide a risk discount could be how the payments differ at the sex act level. In other words, transactional relationships do not typically involve a cash payment negotiated between the woman and the sugar daddy (as it would for FSWs) and therefore the type of payment might be associated with the type of relationship, and thus the likelihood of using a condom. For instance, sex acts where cash does not explicitly change hands, could involve some other implicit exchange (ongoing support, school fees, or trust for relationship building), and these types of sex acts might also be more likely to be unprotected.

We tested the impact of condom use on the likelihood of receiving different types of payment. Table 4 Column 1 shows unprotected sex reduces the chance of receiving anything by 6.0 ppt. A similar impact, 7.3 ppt, as on receiving cash as the payment. Put another way, protected sex increases the chance of a woman receiving something or of receiving cash directly linked to the sex act. This similarity is because 60% of sex acts received anything, and 55% received cash, making the two outcomes highly correlated. There is a small increase in being paid before the sex act of 3.9 ppt. The implication is that the sex act is more explicitly commercial in nature. There is no statistical association with the remaining payment types because instances were rare, so we lack the statistical power to test for this.

**TABLE 4:** Change in likelihood of alternative payment methods following condomless sex acts.

What payment was received:	Anything	Cash	Services	Material support	Paid before
	(1)	(2)	(3)	(4)	(5)
Unprotected sex act	−0.060**	−0.073**	−0.006	0.014	0.039**
	(0.027)	(0.029)	(0.007)	(0.009)	(0.02)
Observations	1886	1886	1886	1886	1886
R‐squared	0.093	0.100	0.013	0.007	0.035
Number of women	744	744	744	744	744
Sex act characteristics	X	X	X	X	X
Client characteristics	X	X	X	X	X
Payment type	n/a	n/a	n/a	n/a	n/a

*Note:* Standard errors are presented in parentheses.

**, ** and * denote statistical significance, at the 1%, 5%, and 10% levels, respectively.

These findings suggest that protected sex might be more akin to commercial relationships and, therefore, more likely to receive cash and more of it, whereas unprotected sex might be reserved for their more regular boyfriends/sugar daddies where they are more invested and therefore demand less payment in general. We explore possible interpretations of these findings further in the discussion.

### 4.2 Premium Modifiers

We test how the premiums change in response to perceived risk of HIV of the male partner through examining the premium attached to unprotected anal sex acts and testing the difference in premiums between women engaging in transactional sex and commercial sex.

As suggested in Table 3, the difference between the price premiums is 36% as per Table 5 column 1. Columns 2 and 3 show that the increased perceived risk of HIV lowers the price premium for FSWs by around 8% for every 10% increase in the perceived chance of the client having HIV, contrary to expectations after controlling for the client's age and perceived wealth, albeit only statistically significant at the 10% level. The higher perceived risk of HIV could be associated with some client characteristics which we are unable to control for, due to limited client characteristics in the data. For instance, clients with a greater perceived risk of HIV, might also exhibit stronger negotiation on prices, or it could be that those clients who negotiate more strongly are perceived to have a higher risk of HIV. For women engaging in transactional sex, there is a small but not statistically significant increase in the premium for sugar daddies that are perceived to have a higher chance being infected with HIV. There exists a large premium for unprotected anal sex for women in commercial sex but not for women in transactional sex.

**TABLE 5:** Premium differences between strata of woman, HIV risk and anal sex.

	Pooled ‐ log of price	Commercial ‐ log of price	Transactional ‐ log of price	Commercial ‐ log of price	Transactional ‐ log of price
	(1)	(2)	(3)	(4)	(5)
Commercial strata * unprotected sex	0.357***				
	(0.132)				
Male partner risk of HIV * unprotected sex		−0.074*	0.015		
		(0.045)	(0.024)		
Anal sex act * unprotected sex				0.596*	−0.067
				(0.361)	(0.251)
Unprotected sex	−0.153	0.513**	−0.201	0.128	−0.134**
	(0.097)	(0.222)	(0.122)	(0.111)	(0.06)
Male partner risk of HIV		0.011	−0.042**		
		(0.023)	(0.017)		
Anal sex act				−0.627***	0.004
(Colorbox)				(0.232)	(0.004)
Observations	3230	2001	1229	2001	1229
R‐squared	0.060	0.065	0.127	0.065	0.116
Number of women	1361	744	617	744	617
Sex act characteristics	X	X	X	X	X
Client characteristics	X	X	X	X	X
Payment type	n/a	n/a	—	n/a	—

*Note:* Standard errors are presented in parentheses.

***, ** and * denote statistical significance, at the 1%, 5%, and 10% levels, respectively.

**FIGURE 1:**
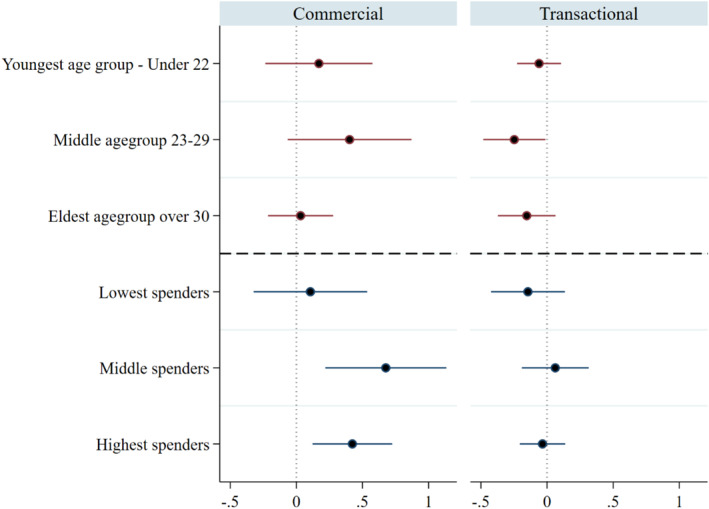
Coefficient plot of risk premium by age group and spending groups. Models estimated using heterogeneous analysis with groups containing around 1/3 of the sample in each. Dots represent point estimates and lines the 95% con.

We explore the difference in premiums by baseline characteristics of age and adult equivalent household expenditure, a proxy for income, split at the 33% percentiles. It appears that both the premium for commercial sex and the discount for transactional are driven by those in their mid‐late twenties with the youngest and oldest showing the least sensitivity to the price they charge for condomless sex, Figure 1. Only for the transactional sex group is this sub‐group analysis still statistically significant at the 5% level. In the commercial sex group, high and medium household spenders accounted for the largest part of the premium, both sub‐groups with a statistically significant at the 5% level and larger premium than in the commercial group. There is less of a pattern in the transactional sex group by expenditure.

### 4.3 Direct Questioning and Other Robustness Checks

To deal with the threat of social desirability bias, we used condom use questions elicited using the *colorbox* method. As a robustness check, we repeat the primary premium analysis using direct questioning about condom use. Contrary to literature that finds results very different between directly questioned and indirect elicitation in observation and quasi‐experimental analysis (Cust et al. 2023; Lépine et al. 2020; Chuang et al. 2021), we find very similar supportive findings, see Table A2 in the Appendix.

Intensity of sex acts can act as a confounder in the relationship between condomless sex and price. Whilst our sex act‐level analysis largely controls away this time‐invariant characteristic, there may be some very short‐term influences of intensity on the premium. We include a “days since the sex act” variable to our models but lose many observations due to outliers and missing information, see Table A3. A second approach is to run a woman fixed effects model using only their most recent sex act at each survey but include wave level indicators of intensity, namely the typical and actual number of patterns and number of sex acts, all in reference to the last 7 days, see Table A5. Both of these approaches lead to similar coefficients but with a loss of statistical power meaning they are no longer statistically significant at the 5% level. We also test our findings using only those who returned for follow‐up therefore excluded all those that might have only one sex act from the data set, finding similar estimates to our main specification, see Table A4.

## 5 Discussion

The risk premium for unprotected sex found for commercial sex is consistent with previous literature. Specifically, the results show that FSWs in Cameroon are paid up to 30% more per sex act by their clients for engaging in unprotected sex. Although this premium is more modest than those found in DRC, Kenya and Bangladesh (Ntumbanzondo et al. 2006; Islam and Russell 2012; Jakubowski et al. 2016), the premium found for FSWs in Cameroon remains consistent with levels found in other LMICs (Gertler et al. 2005; Torre et al. 2010; Arunachalam and Shah 2013). However, our most interesting finding is that this positive premium does not exist amongst women engaging in transactional sex. Our analysis shows these women offer up to a 14% discount for providing unprotected sex to their sugar daddys. The literature hints at a potential discount for “informal sex workers” in Robinson and Yeh's (2011), possibly women engaging in transactional sex. Their pooled analysis finds only a very small premium of 9.3% compared to 24% and 136% premiums found in Kenya among FSWs in other studies (Jakubowski et al. 2016; Manda 2013). Our study helps to reconcile and integrate these previous estimates.

There are several potential explanations for the observed discount. First, it may be that sugar daddies involved in transactional sex have a stronger preference for protected sex compared to clients of FSWs, given that they are often married. If these men are more inclined to engage in safe sex, they might demand unprotected sex less frequently and thus offer lower compensation for it. Second, the women's choice to engage in unprotected sex could be influenced by a lack of awareness regarding the HIV risks, leading them to negotiate inadequate financial compensation for such risky acts. Despite efforts in HIV prevention, such as condom promotion and safe sex awareness campaigns, these initiatives have primarily focused on key populations like FSWs. Women engaging in transactional sex, who do not see themselves as sex workers, may have limited access to these services (Wamoyi et al. 2019). For instance, more than half of the women engaging in transactional sex interviewed for this study had not undergone a HIV test in the previous 12 months, despite their ongoing engagement in transactional sex, such statistics was as high as 83% for FSWs. Alarmingly, many cited not seeing the benefits of testing because they felt healthy, and 65% did not perceive themselves to be at risk of HIV. Third, the premium for unprotected sex might still exist in the transactional sex market, but the way women receive benefits could be different and not fully captured in our data. Finally, transactional relationships are often built on trust and emotional attachment, unlike typical FSW‐client relationships (Wamoyi et al. 2019; Stoebenau et al. 2016). Women in such relationships may be more trusting of their partners and thus less strict about avoiding risky sexual activities. Meeting the desires of their partners might be viewed as a way to demonstrate trustworthiness and invest in the long‐term stability of the relationship, potentially maximizing both material and emotional returns over time. Descriptively supporting this, approximately 50% of the women in transactional sex in our study cited trust in their partners as the primary reason for engaging in unprotected sex.

A key challenge in estimating the causal effect of condomless sex on price is the potential for endogeneity, arising from the joint determination of price and condomless sex. While our fixed effects (FE) model, following the approach of Gertler et al. (2005), effectively accounts for time‐invariant unobserved heterogeneity—such as an individual's risk preferences, negotiation skills, and background characteristics—and sex act and client characteristics used to control for time‐varying confounding, reverse causality remains an issue. Despite being primarily interested in the effect of condomless sex on price, it could be that offering a higher price is leading to a higher likelihood of condomless sex. Both manifest within a negotiation of price and characteristics of the sex act. Without a valid instrumental variable, we are unable to disentangle these competing causes and we appreciate that our estimated effect contains causality running in both directions and should be treated as such. Future research could explore alternative identification strategies, such as leveraging exogenous policy changes or experimental designs, to more robustly establish causality. Nonetheless, our results contribute to the broader understanding of the economics of transactional sex by providing evidence on the pricing dynamics of condom use while acknowledging the methodological challenges inherent in this type of analysis.

Indeed, a key missing variable in the models is whether clients or sugar daddies remained the same or differed between sex acts for participants. Having this information would enable us to estimate a more robust risk premium by accounting for condom use (or lack thereof) with the same client or sugar daddy. Although it may be challenging to implement, if clients or sugar daddies could be identified across women, we could incorporate male partners fixed effects into the models. This would provide deeper insights into how differences among women impact prices and premiums for unprotected sex. In the absence of this, gathering more accurate information about male partners would allow us to explore their characteristics and preferences, potentially revealing variations in demand for condom use.

In the absence of good data on male partners we examine qualitative data collected within this study for evidence. These semi‐structured interviews support some of the hypothesised pathways. A theme within these answers was that male partners preferred using condom to protect their reputation, the implication being the women we were interviewing were secret. For example, one participant said: *“Because most of them are men of reputation and it's not good for their image if it became known that they are with young girls”.* A second theme that shone through was the idea that relationships are built on trust. Respondents would often reference protection being used at the beginning of relationships or demanding STI tests are done before unprotected sex can occur and this demand can come from both men and women. For example, one respondent says: *“To avoid illness, you automatically use a condom. Either that or I demand that you go for a check‐up first and have all the tests done… They accept”*.

## 6 Conclusion and Recommendations

In the last decade, research on the economics of risky sexual behaviours has made significant contributions towards understanding the motivation behind women's involvement in unsafe sex practices and has pointed out financial incentives paid for risky sex services as a key reason. However, these studies have either focused exclusively on FSWs or have conflated transactional sex with commercial sex, overlooking key differences in the supply and demand dynamics of these two markets.

Our study contributes to the growing body of literature on the economics of sexual exchange and is novel in its explicit estimation of the risk premium (or lack thereof) among women engaging in transactional sex. Our study, using a panel of six sex acts, finds a risk premium of around 30% for unprotected sex among commercial sex acts consistent with previous literature of risk premia in LMICs (Gertler et al. 2005; Torre et al. 2010; Arunachalam and Shah 2013). However, most interesting is our finding of a discount of up to 14% for unprotected sex among women involved in transactional sex, a population where the risk premium has not previously been studied. By employing robust fixed‐effects models, we are able to estimate the price change associated with condom use, while controlling for time‐invariant confounders such as risk preferences and other time invariant individual characteristics. These findings suggest that existing theories based on commercial sex are insufficient to explain the dynamics of transactional sex market. We propose that these markets have different features, with men showing distinct preferences for unprotected sex. Additionally, unprotected sex may serve as a strategic investment in the relationship by signalling trust, particularly in times of financial need. Women in transactional sex relationships may also be less informed about the HIV risks they face. Future research should focus on the economic dimensions of transactional sex relationships and the factors driving high rates of unprotected sex and the discount observed for condomless sex in the transactional sex market. Specifically, further investigation is needed into the role and preferences of “sugar daddies,” the intangible benefits sought by women, and the impact of HIV awareness and safe sex education within these dynamics.

Robust policy recommendations are difficult at this stage; however, our findings do support calls to include women engaging in transactional sex to be considered a “key population” in order to receive additional support as FSWs currently do (UNAIDS, 2022). The low level of HIV testing and HIV awareness is worrying, and the lack of HIV risk awareness among women engaging in transactional sex could explain our findings, and further education on HIV would be beneficial. On the other hand, given the high levels of HIV in FSWs in Cameroon (Billong et al. 2019) (albeit not in our sample), interventions that will allow to increase clients preferences for safe sex is needed to reduce the number of sex acts that are unprotected.

## Ethics Statement

Ethics committees at the University College London and the National ethics committee in Cameroon provided ethics approval. Participation was voluntary; respondents gave informed written consent and were reimbursed for their transport costs and time.

## Conflicts of Interest

The authors declare no conflicts of interest.

## Supporting information

Supporting Information S1

## Data Availability

The data that support the findings of this study are available from the corresponding author upon reasonable request.
