# Supplementary material for: Does the Risk Premium Differ Between Women Engaging in Commercial and Transactional Sex? Evidence From Urban Cameroon
Source: Health Econ. 2025 Jun 13;34(8):1474–86. doi: 10.1002/hec.4964 (PMC12212457; doi:10.1002/hec.4964)
Supplement: Supplementary file 1 — Supporting Information S1 [file HEC-34-1474-s001.docx]

# Appendix A

## Design and implementation of the *Colorbox* method

The *colorbox* method involved use of colours and unique PIN codes to elicit participant's responses on their participation in risky sex with their last and penultimate clients or sugar daddies. Before implementation, the interviewers explained what the *colorbox* method entailed to the participants and conducted a practical training phase to ensure they fully understood the method. In addition, during the explanation phase, the interviewers highlighted that the method ensured anonymity with respect to the interviewers as they did not know and could not decode the PIN codes reported by the participants. However, they informed them that researchers were not blinded and could understand responses given.

Table A1 includes the questions asked to both women engaging commercial and transactional sex, all requiring binary responses.

Table A1: Colorbox method questions

| **Women engaging in commercial sex** | **Women engaging in transactional sex** |
| --- | --- |
| “Are you an active sex worker?” | “Are you engaging in sexual relationships with bent necks for which you receive food, cosmetics, clothing, transport, school fees, a place to sleep, alcohol (material support), non-material support (favours) or money?)” |
| “Did you use a condom the last time you had sex with your last client?” | “Did you use a condom the last time you had sex with your last sugar daddy?” |
| “Did you have anal intercourse the last time you had sex with your last client?” | “Did you have anal intercourse the last time you had sex with your last sugar daddy?” |
| “Did you use a condom the last time you had sex with your penultimate client?” | “Did you use a condom the last time you had sex with your penultimate sugar daddy?” |
| “Did you have anal intercourse the last time you had sex with your penultimate client?” | “Did you have anal intercourse the last time you had sex with your penultimate sugar daddy?” |

During implementation, each participant was provided with a bowl of sealed envelopes containing several coupons that would be used to answer the questions asked. Each coupon contained a coloured box and an adjacent 6-digit PIN code separated by a dotted line (Figure *2*). Each code was unique and was only used once among all participants. Once the interviewers read out a question, they followed up with an explanation of what responses the Coloured boxes represented. For instance, if an interviewer asked (“Did you use a condom during sex with your last/penultimate client”), they followed up by stating, (“Black for Yes” and “White for NO”).

The interviewer then asked the participant to select the colour representing their response. After selection, they were then asked to tear the coupon at the dotted line, remain with the coloured box and give only the 6-digit PIN code to the interviewer for data entry. For each question, the participants were asked to pick a new coupon. To ensure confidentiality, the interviewers did not know the PIN codes and what responses they represented. Additionally, they turned away from the participants during the process. This process was repeated for all women engaging in commercial and transactional sex until all responses were collected.


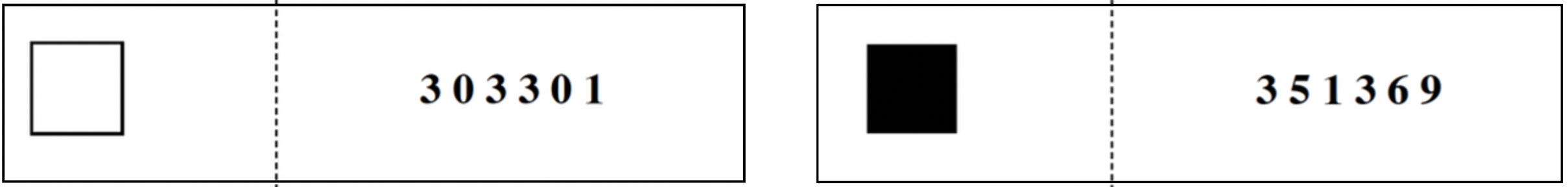


Figure 2: Example of Colorbox method coupons

Table A2. Premium estimates using direct questioning

Table A3: Premiums for women with sex acts within the last 28 days

Table A4: Unprotected sex premium for women who returned for follow up

Table A5: Unprotected sex premium using only the most recent sex act.
